# Improved method for surgical induction of chronic hypertension in mice

**DOI:** 10.1242/bio.059164

**Published:** 2022-07-05

**Authors:** Tom Skaria, Mostafa A. Aboouf, Johannes Vogel

**Affiliations:** Institute of Veterinary Physiology and Zürich Center for Integrative Human Physiology, VetSuisse Faculty, University of Zürich, 8057 Zürich, Switzerland

**Keywords:** Chronic hypertension, Mice model, One-kidney one-clip method, Renal artery clipping

## Abstract

Chronic hypertension can be induced in mice by one-kidney one-clip (1K1C) or two-kidney one-clip surgery, transgenic overexpression of angiotensinogen and renin, administration of deoxycorticosterone acetate-salt, supplying Nitro-L-arginine methyl-ester in the drinking water and Angiotensin-II infusion. Although each model has its own pros and cons, selection of a model that mimics human hypertensive disease accurately is essential to ensure rigor and reproducibility in hypertension research. 1K1C mice represent an efficient, budget-friendly, and translationally capable model; however, their use in preclinical research has remained largely hindered due to concerns about potential technical complexity and lack of reported information regarding procedure-related mortality rates. Here, we describe in detail an improved version of the 1K1C surgery in mice that has zero intraoperative mortality and excellent survival rates in a long-term setting and permits the development of stable chronic hypertension and its target organ complications. Key to this outcome is unilateral nephrectomy 1 week after renal artery clipping to decelerate the blood pressure (BP) increase, which allows the organism to adapt better to the BP rise. The technical and animal welfare improvements presented here may promote the acceptance of the 1K1C model.

## Abstract (precis)

**Summary:** Here, we present the improvements of surgery and post-operative care to minimize intraoperative mortality and to maximize survival rates of the one-kidney one-clip (1K1C) mouse model of human chronic hypertension.

## INTRODUCTION

Chronic primary hypertension (cHTN), a major risk factor for cardiovascular diseases including heart failure (HF) and stroke, affects more than 1 billion adults worldwide (Kearney et al., 2005). The estimated global annual cost of hypertension is $370 billion, approximately 10% of the world's total healthcare expenditure (Gaziano et al., 2009). Therefore, numerous experimental models of hypertension had been developed to aid investigation of the etiology, pathological mechanisms, and effects of therapeutic interventions. The ideal animal model for cHTN should mimic human hypertension and develop its complications in a predictable and controllable fashion, permit interventions during stable states of cHTN and measurements of relevant hemodynamic, cardiac and biochemical variables, and fulfil technical, economical and animal welfare aspects. Alterations in the physiological systems involving neurotransmitters and humoral factors regulating systemic blood pressure (BP), and adverse remodeling of the myocardium and macro- and micro- vasculature causing impaired cardiovascular function are the characteristic features of human HTN that animal models should mimic (Doggrell and Brown, 1998; Lerman et al., 2019).

Systematic analysis of key findings obtained from animal models published in leading scientific journals reveal that only about one-third of them were translated in human randomized trials and only one-tenth of the interventions were finally approved for use in clinics (Hackam and Redelmeier, 2006; van der Worp et al., 2010). An unappreciated challenge and limitation of mouse models in cHTN research, which contributes to translational failure, is that they lack disease progression and chronicity found in human cHTN (van der Worp et al., 2010; Lerman et al., 2019). One-kidney one-clip model (1K1C) of HTN, developed by constricting the renal artery of one kidney with a U-shaped stainless-steel clip and simultaneous removal of the contralateral kidney, shows systemic hypertension accompanied by hypertension-induced end organ damage in mice fed with a standard rodent chow diet (2.5 mg Na^+^/g) (Wiesel et al., 1997, 2001). Previous studies describing 1K1C method as renal artery clipping and nephrectomy performed simultaneously report a mortality rate of 25% within 72 h after 1K1C surgery in mice (Wiesel et al., 2001). We modified the classical 1K1C method of inducing hypertension in mice and found that mice that underwent modified 1K1C developed stable chronic hypertension, and distinct target organ complications comparable to human hypertension such as impaired cerebrovascular reactivity (Wang et al., 2015), and HF phenotypes including eccentric and concentric hypertrophic remodeling and dilated cardiac failure (Skaria et al., 2019). This reveals 1K1C mice as a translationally capable model that accurately mimics human hypertensive disease and its target organ complications. Moreover, the modified 1K1C model allows testing the efficiency of therapeutic interventions for the management of chronic hypertension-induced organ damage such as HF (Skaria et al., 2019). However, the choice of 1K1C mice model in preclinical research remains largely hindered due to concerns about potential technical complexity, and lack of reported information regarding surgery-related intraoperative mortality rates associated with this model that involves multiple major surgeries performed under prolonged general anesthesia. Here, we describe in detail a modified, murine 1K1C surgery with zero intraoperative mortality and excellent survival rates in a long-term setting and development of stable hypertension and its target organ complications. We chose to perform unilateral nephrectomy 1 week after renal artery clipping to decelerate the BP increase, which allows the organism to adapt better to the BP rise. The improvements made in 1K1C technique are mainly centered on minimizing procedure-related trauma to the renal artery and kidney and subsequent ischemic organ damage, decelerating systemic BP rise and intensifying postoperative care. We consider that the technical and animal welfare improvements that fulfil the reduction and refinement imperatives of 3R principle, described in detail in this article may act as resource and increase the acceptance of the modified 1K1C model by the scientific community and thus ameliorate rigor and reproducibility in hypertension research. Moreover, here we present survival rates from larger sample size (*n*=76 mice that underwent modified 1K1C) compared with our previous studies (Wang et al., 2015; Skaria et al., 2019), which may guide researchers to make reliable estimations of mortality rates to be expected in their studies and supply this important information in the mandatory applications for permission to perform animal experiments.

## RESULTS

**Fig. 1.**
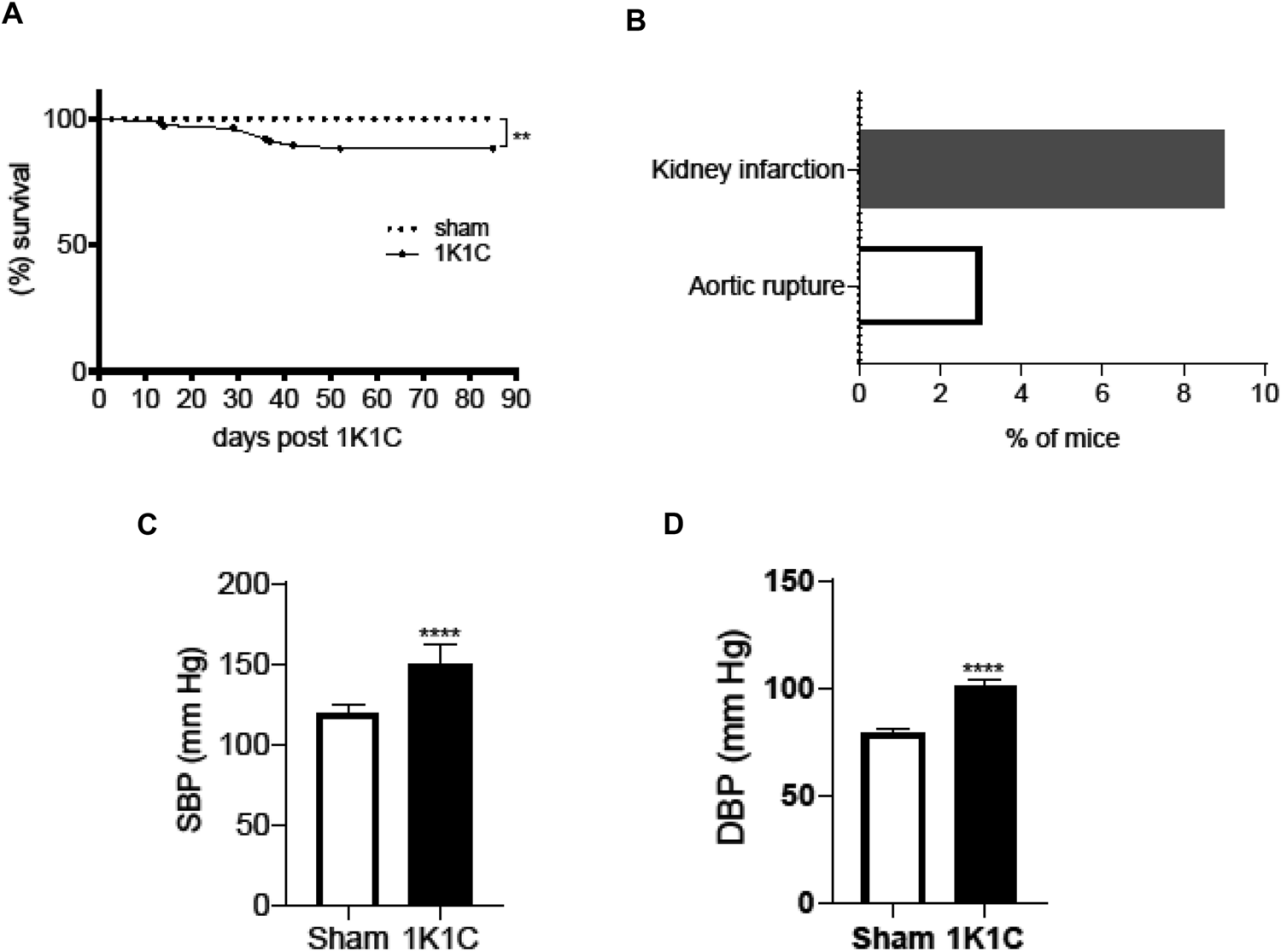
**Outcome of the improved 1K1C procedure (*n*=76).** To generate this figure and Fig. S1, data were taken with permission from our most recent study (Skaria et al., 2019) and statistically compared with that of sham-operated control mice. (A) Kaplan–Meier survival curves of sham-operated (*n*=9) and improved 1K1C-operated (*n*=76) mice. **P<0.01, Mantel–Cox test. (B) Incidences of fatal events diagnosed by autopsy histopathology in 1K1C-operated mice (*n*=76) that were euthanized due to signs of kidney failure or died suddenly without signs of illness post 1K1C surgery. (C) Systolic and (D) diastolic blood pressure (SBP and DBP, respectively) in 1K1C-operated (*n*=7) and their sham-operated control (*n*=9) mice determined by femoral artery catheterization at the end of 12 weeks after 1K1C surgery. Data are means±s.e.m. ****P<0.0001 versus sham; Student's *t*-test.

Mice that underwent modified 1K1C procedure (described in detail in the Materials and Methods section) showed no deaths within 11 days post-nephrectomy (Fig. 1A,B), which is in contrast to previous studies reporting mortality rate of 25% within 72 h after 1K1C surgery (Wiesel et al., 2001). Further, with the improved 1K1C procedure, in a group consisting of 76 mice, only seven mice (9%) were euthanized or died due to kidney infarction (KI, diagnosed as described previously; Skaria et al., 2019) between 12 and 42 days (Fig. 1A,B), which is a significantly improved outcome compared with a previous study reporting lethal KI in 15% (*n*=20) of male C57BL/6 mice within a period of 4 weeks after 1K1C (Wiesel et al., 1997). Two mice (3%) died due to aortic rupture between 37 and 52 days after nephrectomy, the remaining mice (88%) survived up to the study end point (12 weeks after 1K1C) with no detectable signs of impaired well-being (Fig. 1A). No death occurred in the sham-operated group in accordance with previous reports (Wiesel et al., 1997, 2001; Skaria et al., 2019). 1K1C-operated mice that survived up to the study end point (Fig. 1A) showed significantly increased systemic BP [Fig. 1C, D, data were taken with permission from our most recent study (Skaria et al., 2019) and statistically compared with that of sham-operated control mice] and circulating AngII [Fig. S1, data were taken with permission from our most recent study (Skaria et al., 2019) and statistically compared with that of sham-operated control mice]. Further, these chronic hypertensive mice showed significantly increased cardiac hypertrophy (heart weight/tibia length, Fig. S1B), fetal gene reactivation (myocardial mRNA expression levels of Myh6, Myh7 and Nppb, Fig. S1C), and fibrosis (myocardial mRNA expression of Col1a1 and Col3a1, Fig. S1D) (measured as described previously; Skaria et al., 2019) with a decreased cardiac function (ejection fraction; Fig. S1E) as described recently (Skaria et al., 2019) in addition to the previous reports describing enhanced myocardial Nppa mRNA expression post 1K1C (Wiesel et al., 1997).

## DISCUSSION

Animal models have, in general, certain restrictions to mimic human disease that, amongst others, may account for occasional translational failures. Therefore, in preclinical research, animal models of human diseases should be thoroughly reviewed to ensure rigor and reproducibility (van der Worp et al., 2010; Lerman et al., 2019), and maximal meaningfulness.

Previous independent studies revealed that mice subjected to 1K1C surgery develop systemic hypertension and hypertension-induced end organ damage (Wiesel et al., 1997). Our recent studies showed that mice that underwent modified 1K1C develop stable form of chronic hypertension accompanied with activation of pathological processes involved in organ damage (Wang et al., 2015; Skaria et al., 2019), and enable therapeutic interventions for the management of chronic-hypertension induced organ damage such as HF (Skaria et al., 2019).

Pulling the kidney out of the abdominal cavity after detaching it from the surrounding supportive connective tissue and the adrenal gland, and fully exposing it to the outside environment are fundamental steps in widely used protocols for renal artery clipping (Wiesel et al., 1997; Chelko et al., 2012). This may cause significant mechanical trauma to the kidney and exacerbates hypothermia, both are known risk factors for adverse outcomes after major abdominal surgery (Máca et al., 2018).

We have introduced modifications to the standard 1K1C protocol to reduce its mortality that are mainly centered on the following five critical factors; (i) prevention of renal artery twisting or angulation/kinking, (ii) minimization of procedure-related trauma and ischemic damage on the kidney, (iii) deceleration of the surgery-induced blood pressure rise, (iv) reducing the duration of a single session general anesthesia, and (v) intensified post-operative care. It is essential to prevent artery twisting during clipping because cHTN itself is a well-established cause for vessel tortuosity that in turn leads to thrombosis and ischemic attacks in distant organs, which may be further exacerbated by clipping-induced artery tortuosity. Vessel remodeling involving elastin fiber fragmentation and reduced axial stretch lead to high prevalence of vessel tortuosity in hypertensive patients, and numerous studies suggest vessel tortuosity as an indicator of arterial hypertension, stroke and ischemic heart disease (Han, 2012). Moreover, retaining the kidney in its original position during clipping, another modification of 1K1C technique described in detail here, prevents organ movements after surgery and reduces the risk of bending the renal artery. Renal artery bending could result in kidney ischemia or thrombosis at the bended vessel section due to the induction of turbulent blood flow. Besides minimizing the development of ischemic damage by preventing artery tortuosity, direct mechanical trauma to the kidney (the artery of which to be clipped) resulting from disruption of kidney-renal fascia connective tissue interaction, lifting the kidney out of abdominal cavity and exposing it fully to the environment during clipping was avoided by fine-tuned artery clipping technique (described in detail in the Materials and Methods section). Renal fascia anchors the kidneys to surrounding structures to prevent bumps and jolts to the body from injuring the kidneys (Brant and Helms, 2007). Other critical aspects we refined are the long duration of single session major surgery and general anesthesia. Longer (for example, 45 min), in contrast to shorter (for example, 20 min) period of general anesthesia during a single exposure leads to hypothermia, hypoglycemia and disturbances in cardiac rhythm and may adversely affect the post-operative recovery in rodents (Gargiulo et al., 2012; Kikuchi et al., 2013; Hohlbaum et al., 2017). Further, longer abdominal surgery, or prolonged general anesthesia, may trigger hypoxic signaling causing activation of local and systemic immune pathways affecting wound repair (Yang et al., 2017; Máca et al., 2018). Therefore, splitting the surgery into two sessions of 1 week apart, another modification of 1K1C technique described in detail here, allows the mouse to adapt better to the sudden increase in systemic BP. This is not the case with the classical 1K1C procedure involving simultaneous renal artery clipping and nephrectomy (Wiesel et al., 1997, 2001). With the improved 1K1C method, during the first week, the mice are actually subjected to the 2K1C model that is known to increase BP in a lesser degree compared with classical 1K1C procedure. Thus, with the improved 1K1C procedure, the mice can cope much better with further increase in BP when removing the non-clipped kidney. Moreover, this modification also allows better diagnosis of failure of the clipped kidney since removal of the kidney itself is very well tolerated (cf. sham animals). After the second surgery (nephrectomy), any symptoms of reduced wellbeing of the mice during the first 3 days can be accounted to failure of the remaining, clipped kidney. Lastly, in contrast to the classical description of first few hours post-surgery (until they have recovered from anesthesia and are ambulatory) in widely used protocols (Bernal et al., 2009), an intensive post-operative care (maintaining the mice at 32°C for an extended period and providing them with soft food) was adopted to support the mice during recovery period. This is because compared with other laboratory animals used in hypertension research, mice have larger surface area to body ratio and consume substantial amount of energy solely for sustaining their body temperature. In mice, the thermoneutral zone (temperature at which organisms spend minimum amount of energy) is 29–33°C. A fall in temperature below this range increases activity of sympathetic nervous systems to enhance thermogenesis to a level that preserves body temperature. Moreover, decreased temperature affects systemic BP. Evidence indicates about 1.6 mm Hg increase in BP for each 1°C loss in ambient temperature between 18 and 30°C. Therefore, keeping the mice in a warm environment during the first three post-operative days allows them to better adapt to the fast increase in BP especially during switching from the 2K1C to 1K1C model. Thus, minimization of heat loss and heat production and the improved post-operative care may sufficiently contribute to the survival during the post-surgical period in 1K1C mice (Van Vliet et al., 2006).

A modified 2K1C procedure was reported in which each individual attempt to constrict the renal artery required manual slicing of a small segment of polyurethane tubing of fixed internal diameter (0.30 mm) lengthwise to produce a cuff followed by placing the cuff around the renal artery and manually closing the gap of the cuff with a silk ligature (Lorenz et al., 2011; Saleem et al., 2020), thereby raising the possibility of operator-dependent variations in the internal diameter of cuff. Of note, this procedure is, per se, more sophisticated and consequently requires longer anesthesia time. Moreover, the hypertensive heart of 2K1C mice, in contrast to that of human and 1K1C mice (Wiesel et al., 1997; Kessler-Icekson et al., 2002), exhibits unchanged expression of atrial natriuretic peptide (ANP; regulator of fluid homeostasis and BP) (Wiesel et al., 1997).

The improved survival rates from quite large sample size (*n*=76) in this report may guide researchers to make reliable estimations of mortality rates to be expected in their studies and supply this important information in the mandatory applications for permission to perform animal experiments. This study was performed according to the 3R principles and regulations of Cantonal Veterinary Department, Zurich, Switzerland, in reducing the number and suffering of animals used in research. Therefore, we could not get the permission from the authorities to further characterize the contribution of each, individual modification to the observed overall improvement in survival of our mice.

Taken together, the improved 1K1C surgery has zero intraoperative mortality and thus prevents wastage of animals. Further, the improvements presented in detail in this article reduce the acute (72 h) post-operative mortality in a chronic setting, improve the technical and animal welfare considerations of the translationally capable 1K1C mice hypertensive model, and will guide scientists to broader utilization of this model in the preclinical research.

## MATERIALS AND METHODS

Male C57BL/6J mice, aged 14–16 weeks, kept in T2 standard rodent cages with sawdust bedding and free access to drinking water and standard food (Kliba No. 3430, Provimi Kliba), maintained at 21±1.0° C in a climatically controlled environment on a 12 h light/dark cycle were used for the experiments (Skaria et al., 2019). All experiments were performed in accordance with the ‘European Convention for the Protection of Vertebrate Animals used for Experimental and other Scientific Purposes’ (Council of Europe No. 123, Strasbourg 1985) and institutional and local governmental guidelines, and were approved by the Cantonal Veterinary Department, Zurich, Switzerland.

### Renal artery clipping

**Fig. 2.**
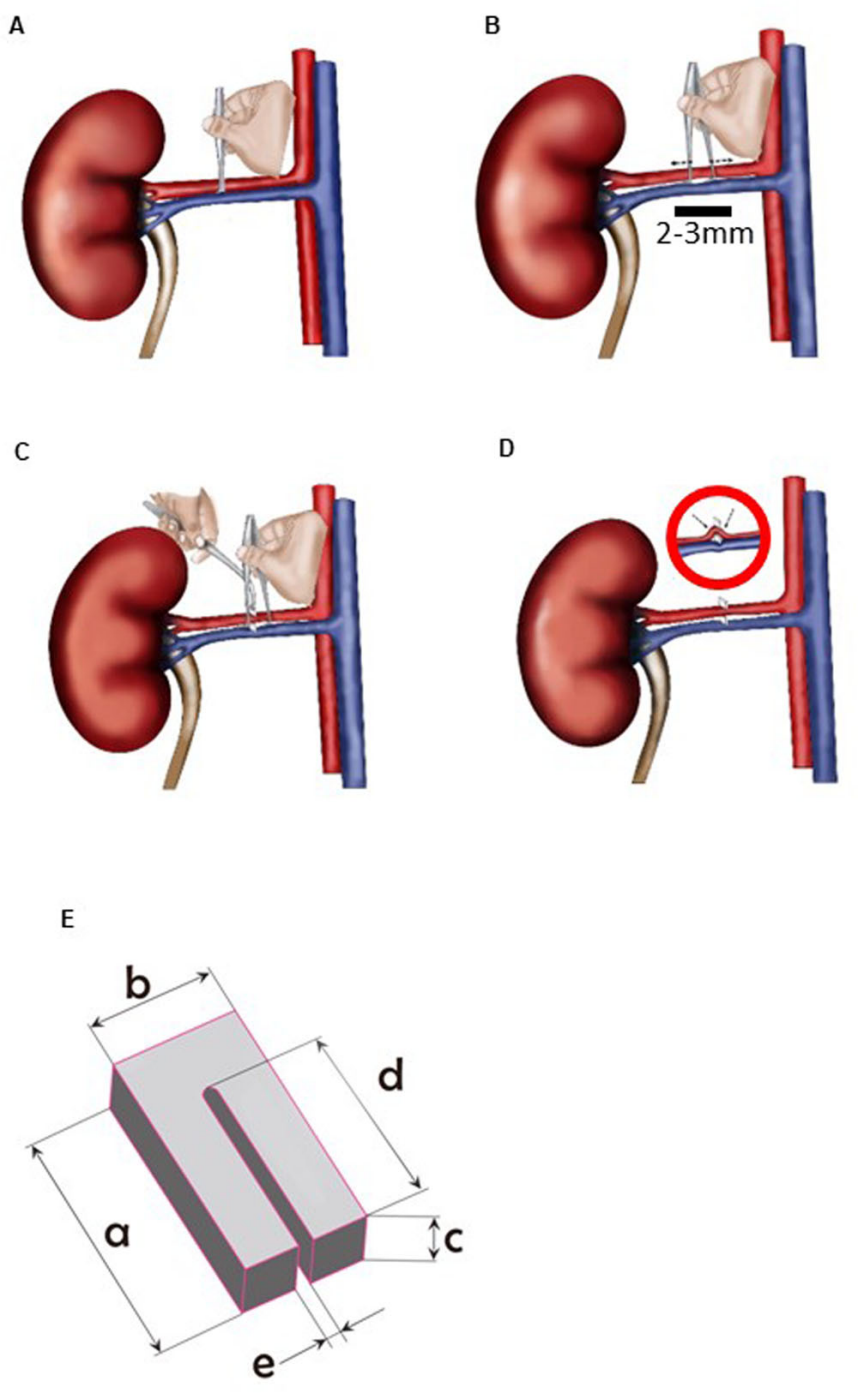
**First surgery for the improved 1K1C procedure (clipping the left renal artery).** The kidney stays *in situ* and renal vessels are exposed. (A) Positioning of closed, serrated forceps between the renal artery and vein and (B) opening of the forceps handles to move the serrated jaws apart (dashed arrows indicate movement of forceps ends in opposite direction, leading to separation of renal artery from vein). This procedure (A,B) must be repeated three to four times. (C) Once the vessels are separated over a length of 2–3 mm, the artery is lifted gently with the serrated forceps and the metal clip (hold with left hand) is slipped carefully over secured artery (hold with right hand). (D) Proper renal artery clipping with the improved procedure does not lead to angulation, kinking or twisting of the artery at both sides of the metal clip. It is important to check this under the microscope. Inset image in panel D shows unfavorable clipping with vessels separated at an insufficient length and angulation/kinking of artery. Neither adrenal gland nor the attachments of the kidney with the surrounding tissue are manipulated, hence these structures are not shown here. (E) Technical drawing and dimensions of the stainless-steel surgical clip used (Exidel SA R&D Microtechniques). a: 2.00 mm, b: 1.00 mm, c: 0.80 mm, d: 1.50 mm, e: 0.12 mm. The edges of the clip are slightly rounded off, which is not depicted in the figure.

Mouse was weighed and premedicated with Buprenorphine, 0.1 mg/kg, s.c. General anesthesia was induced with 5% isoflurane in O_2_ and thereafter maintained with 2.5% isoflurane in O_2_. After applying lubrication gel (Viscotears, Bausch-Lomb) to both eyes, mouse was placed on its right side (left kidney facing up) on a heating pad maintained at 37°C, and the limbs were positioned and fixed with adhesive tape. The skin above left kidney was wetted and shaved using a scalpel blade to remove the fur from this area. Shaved area was then wiped with 70% ethanol using cotton swabs. The skin above left kidney was pulled up using sterile Graefe (cat. 14141-G, WPI) or Micro dissecting Iris (cat. 504478, WPI) forceps, and a small incision, approximately 5–7 mm long, was made into the skin and abdominal wall (above the left kidney) with a sterile dissecting scissor (cat. 500367-G, WPI). Quite rarely, some blood needed to be removed using sterile cotton swabs. Then, the cut skin and abdominal muscle layers were retracted using Rochester-Carmalt Hemostatic forceps (cat. 501243-G, WPI), and the kidney and renal vessels were located through a dissection microscope. Pulling the kidney out of the abdominal cavity after detaching it from the surrounding supportive connective tissue and the adrenal gland, and fully exposing it to the outside environment are fundamental steps in widely used protocols for renal artery clipping (Wiesel et al., 1997; Chelko et al., 2012). This may cause significant mechanical trauma to the kidney and exacerbates hypothermia, both are known risk factors for adverse outcomes after major abdominal surgery (Máca et al., 2018). Moreover, detaching the kidney from the surrounding connective tissue may cause uncontrolled movement of the organ in the abdominal cavity after surgery which could bend the supplying vessels and thus increase the risk of ischemia. To avoid this, we maintained the kidney with its intact connective tissue attachments in its original position in abdominal cavity during the whole clipping procedure. Surgical grade absorption sponges (triangle shaped, unmounted; cat. 18105-03, F.S.T) were then used to remove thin layers of fat surrounding the renal vessels. The serrated jaws of a sterile Dumont #5B forceps (cat. 500234, WPI), held in closed position (with pressure applied to hold the shanks together), were positioned between the renal artery and vein (Fig. 2A), followed by slowly opening the forceps to move the serrated jaws apart (Fig. 2B).

This process was repeated two to three times until clear separation of renal artery from vein over an area of approximately 2–3 mm length was achieved. This ensured that the renal artery to be clipped was separated from the vein for a distance as long as possible (Fig. 2A,B). This modification prevents too tight positioning of metal clip at the narrow space between renal artery and vein, and consequently twisting and/or bending of the constricted artery at the edges of the metal clip, which may lead to severely impaired perfusion and ischemic organ damage (Han, 2012). The separated artery was cleaned using sterile, triangle shaped surgical grade absorption sponges to remove fragments of connective tissue and fat. While having the separated part of renal artery (that is to be clamped) securely positioned upon the serrated jaws of Dumont #5B forceps, using Dumont #7 forceps (cat. 14079, WPI), the closed end of the sterile, U-shaped stainless-steel surgical clip (2×1×0.8 mm outer dimensions with a gap width of 0.12 mm and gap length of 1.5 mm, Part No. CHUV-00.000.012, Exidel SA R&D Microtechniques; Fig. 2E) was grabbed and the clip's open end was slipped over the secured renal artery (Fig. 2C; Fig. S2). Forceps were withdrawn, and abdominal cavity surrounding the clamped area was cleaned using sterile, dry cotton swabs. After suturing the muscle (6-0 Vicryl, cat. V926H, Ethicon) and skin (7-0 Polyamid, cat. EH7446H, Ethicon) individually with single-button sutures, the surgical wound was cleaned using sterile, dry cotton swabs. Analgesia was given in the form of Flunixin (diluted in a sterile solution containing 0.9% NaCl and 5% D-glucose and administered 5 mg/kg s.c., cat. 5212-600 C, Graeub). Anesthesia was then terminated. Eye lubrication gel was reapplied. When the mouse was fully awake and resumed its movements, it was transferred to a ventilated, clean, dry post-operative chamber (with clean un-shredded tissue paper bedding instead of wood chips to reduce the risk of tracheal obstruction) maintained at 37°C and incubated there for 1 h during which it was provided with softened food (pelleted diet soaked in 5% glucose solution). Mouse was then returned to its cage (with softened food and drinking water) placed in a ventilated, clean chamber maintained at 32°C and incubated there for 3 days. This intensified post-operative care involving modified temperature conditions over an extended period of 3 days, in contrast to the classical description of first few hours post-surgery (until they have recovered from anesthesia and are ambulatory) in widely used protocols (Bernal et al., 2009), may maintain mice in their thermoneutral zone and thus prevent any unintended hypothermia following long abdominal surgery, and save energy for the animal during post-operative recovery period (Van Vliet et al., 2006). Mouse was then transferred to the standard housing conditions. Analgesia (Flunixin, cat. 5212-600 C, Graeub; 5 mg/kg in sterile 0.9% NaCl solution, warmed to 37°C, and administered s.c.) twice daily was continued for 3 days after surgery, and mouse was monitored daily for recovery and wellbeing.

### Nephrectomy

**Fig. 3.**
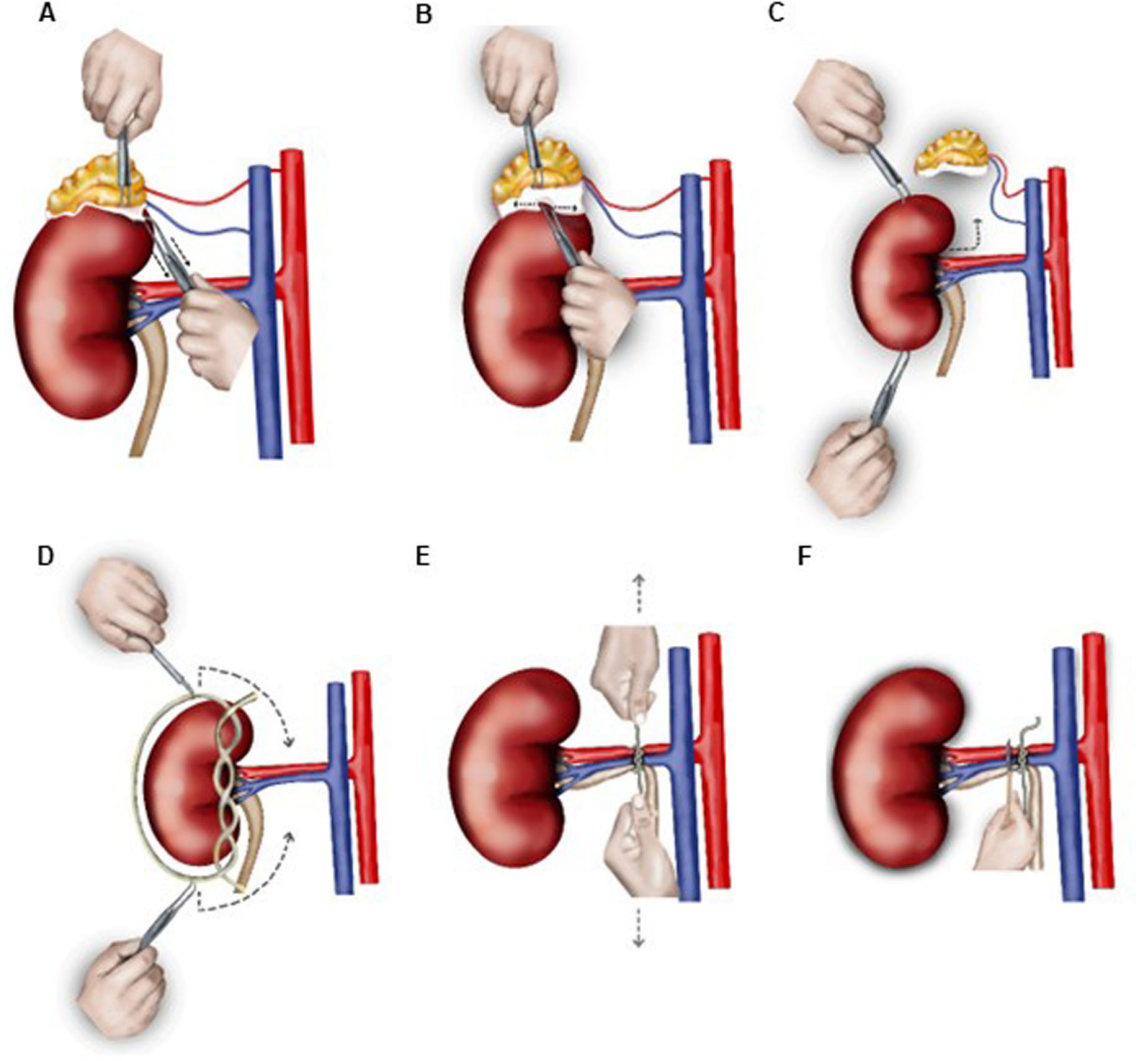
**Second surgery for the improved 1K1C method (nephrectomy of the right kidney).** (A) Retracting the adrenal gland with curved forceps in left hand and pulling down the connective tissue and fat layer attached to the cranial kidney pole with curved dissecting forceps in right hand (dashed arrows indicate downward movement). (B) Forceps in right hand is then slowly open to move the forceps' serrated jaws apart (indicted by dashed arrows). Repeating this step results in disruption of the connective tissue attachment between kidney and adrenal gland. (C) The kidney is gently lifted out of the abdominal cavity using forceps without squeezing the organ. Note that squeezing the organ with the forceps can induce damage of the kidney capsule and bleeding (translocation is indicated by dashed arrows). (D) A loose ligature (surgeon's knot type) is placed over the kidney and slipped down using forceps (dashed arrows indicate direction of slipping down aided by forceps around the kidney, its vessels and the ureter). (E) Renal vessels and ureter are tied off/obliterated (dashed arrows indicate tightening the knot by pulling apart with left and right hands in opposite direction). (F) Kidney is excised above the tied-off suture using a scalpel or round-tipped scissor.

We chose to perform unilateral nephrectomy 1 week after renal artery clipping to decelerate the BP increase, which allows the organism to adapt better to the BP rise. This is not the case with the classical 1K1C procedure involving simultaneous renal artery clipping and nephrectomy (Wiesel et al., 1997, 2001). One week after renal artery clipping, the mouse was premedicated and anesthetized as described above for clipping. After lubricating the eyes, mouse was placed on its left side (right kidney facing up) on a heating pad maintained at 37°C and limbs and skin above the kidney were prepared as described above for clipping. Using a sterile dissecting scissor, a small incision, approximately 7 mm long, was made into the skin and abdominal wall above the right kidney. The cut skin and abdominal muscle layers were secured using retraction forceps, and the kidney and renal vessels were located through a dissection microscope. Using the ends of sterile Graefe (cat. 14141-G, WPI) or Micro dissecting Iris (cat. 504478, WPI) forceps, the kidney was gently separated from fat and adrenal gland (Fig. 3A,B) and was then lifted out of abdominal cavity (Fig. 3C). Of note, the tearing effect of forceps was applied only upon the fat/connective tissue, and touching or grabbing the adrenal gland with forceps was avoided to minimize tissue injury during the procedure (Fig. 3A,B). Renal vessels and ureter were ligated with silk suture material (4-0, Silk, cat. EH6802H, Ethicon) using a surgeon's knot (two throws of suture on the first tie and one throw opposite direction in second tie; Fig. 3D,E). Thereafter, using sterile scalpel or dissecting scissor, the kidney was excised above the tied-off suture (Fig. 3F, Fig. S2). The surgical area was cleaned, muscle and skin were sutured, and post-operative care including analgesia were provided as described above for clipping. Care was taken to avoid resting the surgeon's hands or surgical instruments on the chest or abdomen of anesthetized mice throughout the procedure as the external pressure may interfere with the blood flow and respiration. Further, the kidney must not slide back once lifted out of the abdominal cavity. A sham operation consisted of the whole surgery except placing the clip around the left renal artery (Wiesel et al., 1997; Skaria et al., 2019). All modifications and improvements made to the 1K1C method for establishing chronic hypertension in mice are listed in Table 1.

**Table 1.**
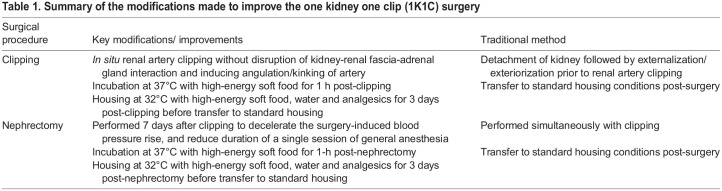
Summary of the modifications made to improve the one kidney one clip (1K1C) surgery

### Statistical analysis

Data were analyzed using GraphPad Prism software version 5.0f (GraphPad Software, San Diego, CA, USA), and tested for normality by Kolmogorov–Smirnov test. When parametric assumptions were satisfied, two independent groups were compared using an unpaired Student's *t*-test. Survival data were plotted using the Kaplan–Meier method and the comparison was conducted using the log-rank (Mantel–Cox) test. Differences were considered statistically significant at *P*<0.05.

## Supplementary Material

Supplementary information
