# Supplementary material for: Improved method for surgical induction of chronic hypertension in mice
Source: Biol Open. 2022 Jul 5;11(7):bio059164. doi: 10.1242/bio.059164 (PMC9277079; doi:10.1242/bio.059164)
Supplement: Supplementary information [file biolopen-11-059164-s1.pdf]

Fig. S1

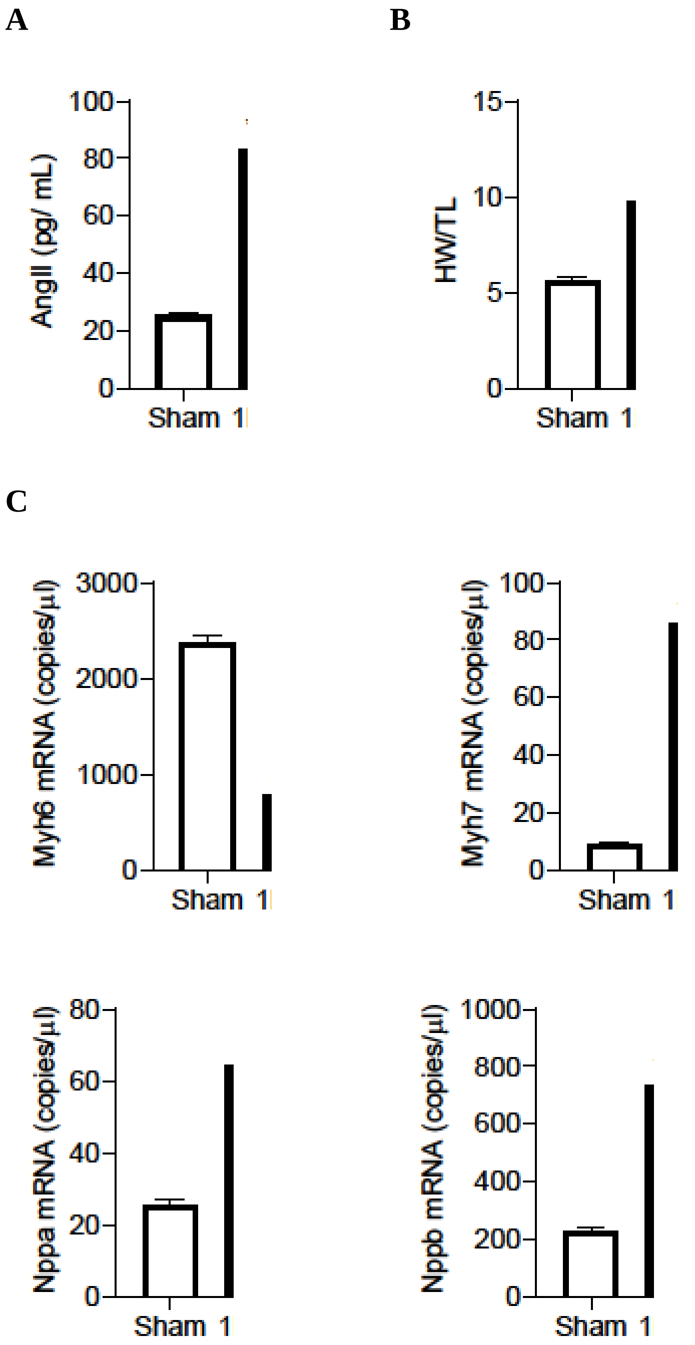

Biology Open • Supplementary information

D

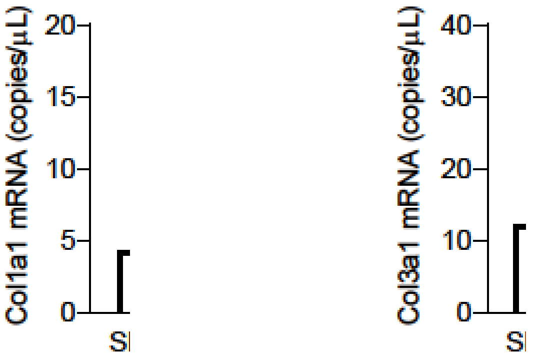

E

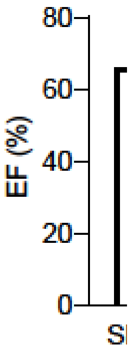

Fig. S1. Data related to systolic and diastolic blood pressure (SBP and DBP respectively), plasma Angiotensin-II (AngII) concentration, cardiac hypertrophy (indicated by heart weight/tibia length; HW/TL), gene expression signature of fetal gene reactivation (mRNA expression level of Myh6, Myh7, Nppa, Nppb), fibrosis (mRNA expression of Collagen 1 and Collagen 3; Col1a1 and Col3a1 respectively), and cardiac function (ejection fraction; EF) in 1K1C-operated chronic hypertensive vehicle-treated C57BL/6J mice and respective sham-operated mice were taken from (Skaria et al., 2019) after obtaining permission from publisher of previous study and statistically analysed to generate Fig. S1. (A) plasma AngII concentration (determined by ELISA), (B) HW/TL, (C) myocardial mRNA expression levels of Myh6, Myh7, Nppa, and Nppb (determined by droplet digital PCR), (D) myocardial mRNA expression of Col1a1 and Col3a1 (determined by droplet digital PCR), and (E) EF (determined by echocardiography) in 1K1C-operated chronic hypertensive (n = 7) and their sham-operated control (n = 9) mice. Data are means ±S.E.M. **P<0.01, ****P<0.0001 vs Sham; Student's t test.

Biology Open • Supplementary information

Biology Open (2022): doi:10.1242/bio.059164: Supplementary information

Figure S2

A

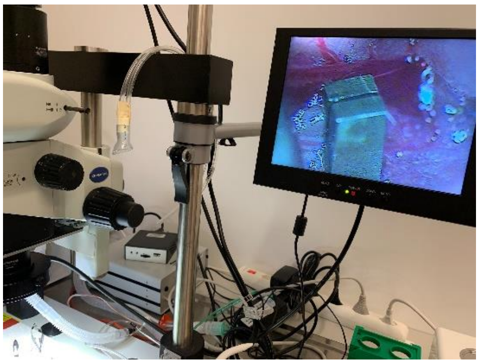

B

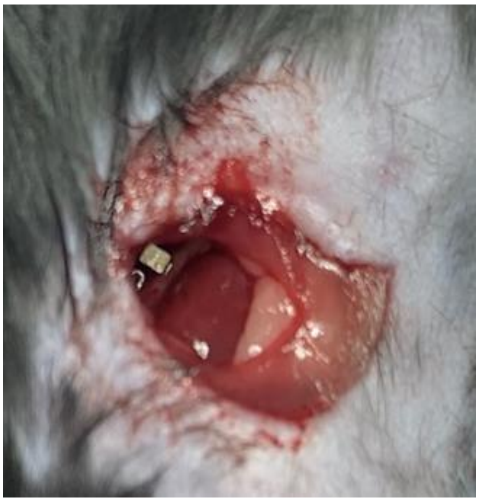

C

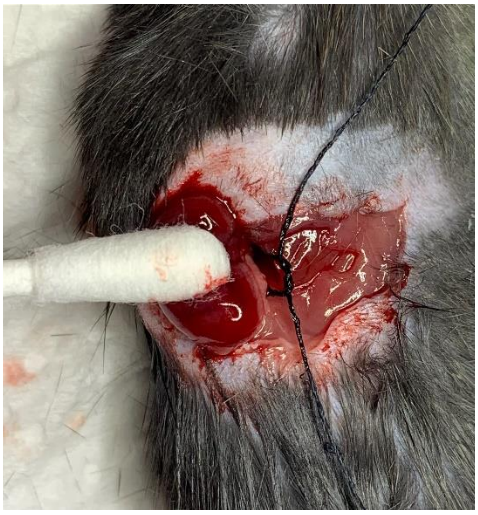

Fig. S2. Original images of clipped renal artery (A, B) and placement of a loose ligature (surgeon’s knot type) over the kidney and slip down prior the renal vessels and ureter are tied-off as part of nephrectomy, following the actual, modified 1K1C procedure.

Biology Open • Supplementary information

References

Skaria, T., Mitchell, K., Vogel, O., Walchli, T., Gassmann, M. and Vogel, J. (2019). Blood Pressure Normalization-Independent Cardioprotective Effects of Endogenous, Physical Activity-Induced Alpha Calcitonin Gene-Related Peptide (alphaCGRP) in Chronically Hypertensive Mice. *Circulation research*.

Biology Open • Supplementary information
